# Advances in thin layer deposition techniques in perovskite solar cells

**DOI:** 10.1039/d5ra05921f

**Published:** 2025-10-22

**Authors:** Qamar Wali, Nahin Ar Rabbani, Sadia Afrin, It Ee Lee, Muhammad Yar Khan, Muhammad Aamir

**Affiliations:** a Centre for Smart Systems and Automation, COE for Robotics and Sensing Technologies, Multimedia University 63100 Cyberjaya Malaysia ielee@mmu.edu.my; b Faculty of Artificial Intelligence and Engineering, Multimedia University 63100 Cyberjaya Malaysia; c Department of Chemistry, North Carolina A&T State University Greensboro NC USA; d Department of Physics, Qilu Institute of Technology 250200 Jinan Shandong Province P.R. China; e Department of Chemistry, Mirpur University of Science and Technology (MUST) Mirpur-10250 (AJK) Pakistan

## Abstract

Perovskite solar cells (PSCs) have shown excellent performance in the photovoltaic field, with the highest power conversion efficiency (*η*) reaching 27%. This significant achievement was possible due to the optimization of various active layers, such as the perovskite light absorber and charge transport layers. Various defects arise during the deposition of thin-film layers, such as defects in the bulk or surface and at the perovskite/electron transport layer (ETL) or perovskite/hole transport layer (HTL). These defects act as a catalyst and trigger the degradation of PSCs. The non-radiative recombination caused by these defects significantly lowers the open circuit voltage, fill factor and current density of the device. Various techniques have been established to develop pinhole-free and compact layers that could significantly improve the crystallinity of the perovskite and the ETL or HTL, thereby reducing the density of defects. In this review, we provide an overview of recent advances in the synthesis of perovskite materials using diverse techniques such as vapour deposition, spin coating (single and double steps), and hot casting. To our knowledge, the hot casting and spin coating approaches yield high photovoltaic parameters.

## Abstract 2

Perovskite solar cells (PSCs) have shown excellent performance in the photovoltaic field, with the highest power conversion efficiency (*η*) reaching 27%.

## Graphical abstract

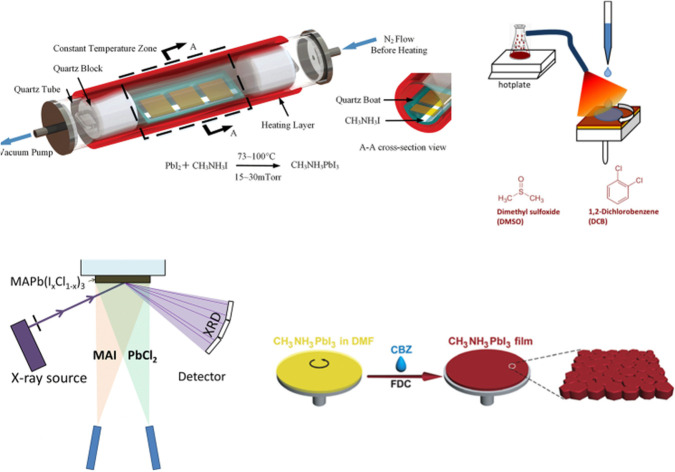

## 1 Introduction

Perovskite solar cells (PSCs) have rapidly become a leading candidate for next-generation photovoltaics because they combine high power conversion efficiency (*η*) with the potential for low-cost fabrication.^1–4^ The impressive gains in PSC performance arise from the favourable optoelectronic properties of the halide perovskites, such as strong light absorption, long carrier diffusion lengths, tunable bandgaps and relatively high defect tolerance, together with the continued optimization of the active layers and charge transport materials.^1,5,6^ While this field was initially dominated by hybrid organic-inorganic materials, significant research efforts are now also directed towards all-inorganic perovskites, such as those based on cesium (Cs^+^), in an effort to overcome the inherent thermal instability of the organic cations. However, widespread deployment is held back by durability and reproducibility issues that are closely tied to film quality and defect formation during fabrication.^7^ Defects can occur in the bulk perovskite, at the film surface, or at the interfaces with the electron transport layer (ETL) and hole transport layer (HTL); these defects act as traps for charge carriers and increase non-radiative recombination, thereby reducing the open-circuit voltage (*V*_oc_), short-circuit current (*J*_sc_) and fill factor (FF), which together limits both the initial *η* and stability.^7–9^ Because defect density and distribution are so strongly influenced by how the perovskite and transport layers are deposited, the choice and control of the deposition methods are central to improving both device performance and stability.^1,10–12^ Historically, manual lab-scale methods such as spin coating dominated early progress due to their simplicity and the fine control they have for small-area devices. However, these methods suffer from poor material utilization, limited scalability and batch-to-batch variability, which complicate reproducibility and large-area manufacturing.^11,13^

To overcome these limitations, the field is moving toward scalable, high-throughput techniques that can produce uniform, pinhole-free films across larger areas. The prominent examples include slot-die and other roll-to-roll compatible wet-coating methods, controlled vapor-phase deposition and hybrid approaches that combine the solution and vapor steps.^10,14,15^ Slot-die coating and related continuous solution processes are attractive because they translate directly to industrial roll-to-roll lines and can deposit uniform layers at high speed when the ink formulation and process window are well optimized.^15^ Vapor-based routes, and in particular, mediated vapor deposition strategies, provide exceptional control over film stoichiometry, thickness and uniformity and have been shown to yield high-performance modules with improved reproducibility, making them promising for scale-up.^16–18^ Hybrid layered approaches that blend the advantages of solution and vapor processing have also demonstrated improved crystallinity and lowered trap densities, producing films that combine large grain size and compact morphological features that reduce the pathways for environmental ingress and non-radiative loss.^19^ Alongside the choice of deposition technique, progress in automating laboratory methods has improved repeatability and data reliability. Fully automated spin-coating and controlled quenching systems reduce human variability and deliver more reproducible small-area devices, which aid in systematic study and in the transfer of recipes to scalable platforms.^13^ Equally important are post-deposition and interface treatments that complement deposition strategies like targeted passivation, surface functionalization, and plasma-based treatments, which can heal bulk and surface defects, reduce trap states and improve interfacial contact between the perovskite and charge transport layers, thereby mitigating recombination and enhancing stability.^7,11^ Because device architecture and material choice (planar *vs.* mesoporous, different ETL/HTL chemistries) influence the ideal deposition route, many recent efforts emphasize the co-design of material systems and deposition workflows so that processing yields the desired film microstructure and energetic alignment at the interfaces.^1,5,11^

Practical deployment also requires that the deposition methods be compatible with large-area module manufacturing without compromising the film quality required for high *η*. To achieve this balance, comparative studies of spin coating, hot casting, slot-die and various vapor approaches are needed to identify the process parameters that control grain growth and defect formation during crystallization.^10,14,15,19^ Hot casting and optimized spin-coating variants have repeatedly produced perovskite films with larger grains and fewer boundary defects in lab-scale studies.^13,19^ Nevertheless, scaling those successes to meter-scale modules requires attention to solvent drying dynamics, precursor formulation, substrate temperature control, and inline monitoring aspects that are actively addressed by scalable deposition research summarized in the literature.^10,14^ Improving the *η* of PSCs and their stability is not simply a matter of material discovery but depends on mastering deposition science, selecting and tuning the deposition routes, integrating hybrid and post-treatment steps, and aligning process design with device architecture, which can minimize defect formation, suppress non-radiative recombination, and yield reproducible, durable devices suitable for industrial manufacture.

**Fig. 1:**
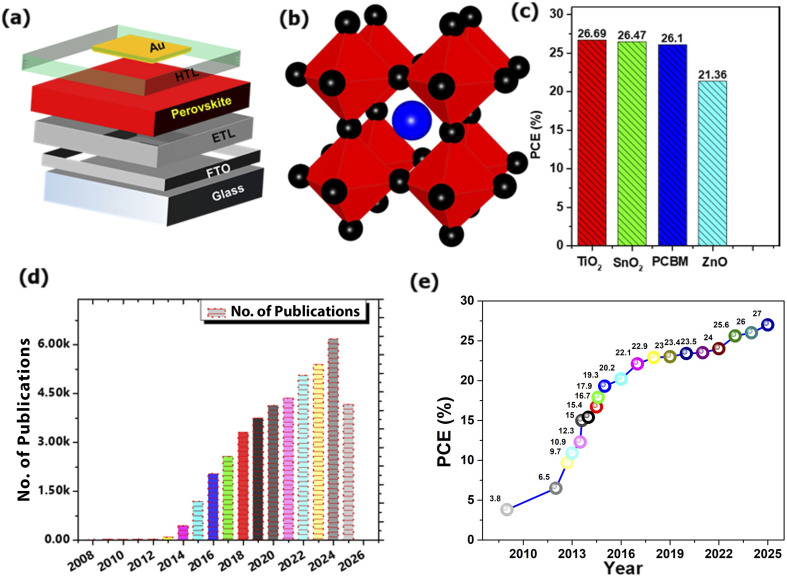
(a) Schematic diagram of a regular n-i-p PSC. (b) Crystal structure of a PSC. (c) *η* comparison of various ETLs (d) statistics of publication for PSCs (data taken from Scopus, dated 10 Aug 2025) and (e) progress in *η* of PSCs, adapted/reproduced from ref. 20 with permission from NREL, Best Research-Cell Efficiency Chart, copyright 2025.

A schematic of a regular n-i-p PSC is illustrated in Fig. 1a. The crystal structure of the PSC is illustrated in Fig. 1b. This structure is characterized by a cubic arrangement where a large organic or inorganic cation (represented by the central blue sphere) is in the centre, smaller metal cations (at the centre of the red octahedron) occupy the corner sites, and a halide anion (shown as the black spheres) occupies the face centres A comparison of the *η* values of PSCs utilizing different materials for the ETL is illustrated in Fig. 1c. This highlights that TiO_2_-based devices achieve the highest *η* of 26.69%, outperforming those with SnO_2_ at 26.47%, PCBM at 26.1%, and ZnO at 21.36%. The bar graph in Fig. 1d illustrates the exponential growth in scientific interest in PSCs. The trend shows a dramatic increase in research output, indicating a highly active and rapidly evolving field. The record-breaking advancement in the *η* of PSCs over time, from 2009 to early 2025 is shown in Fig. 1e. Starting from an initial efficiency of 3.8% in 2009, the certified *η* has rapidly climbed to 27%.^20^

This review aims to provide a comprehensive and systematic overview of the advanced deposition techniques by surveying recent advancements in perovskite deposition techniques, transitioning from lab-scale methods like spin coating to scalable approaches such as slot-die coating and vapor deposition. The choice of deposition method significantly impacts the perovskite film's morphology, defect density, and interface quality, which in turn govern device performance and long-term stability. Strategies, including passivation techniques, are discussed as effective means to mitigate bulk and interfacial defects. This review identifies key challenges in translating laboratory success to commercially viable modules and outlines future research directions to accelerate this transition. Ultimately, this work provides a comprehensive overview of the state-of-the-art in perovskite deposition, offering a forward-looking perspective on the most promising pathways to unlock the full potential of perovskite technology for next-generation solar energy.

## 2 Deposition techniques of perovskite materials in PSCs

The morphology of perovskites has a significant impact on the overall performance of PSCs. A high-quality thin film is essential for PSCs as well as other optoelectronic devices. The fabrication of perovskite thin films has been evaluated and investigated during the past several years with incredible progress; however, a review of the published literature indicates that the growth dynamics of perovskite films are relatively less understood. It is critical to gain a deep insight into the perovskite film growth mechanisms to enable the use of different film fabrication procedures. In general, two major types of methods are used in the synthesis of perovskite thin films, *i.e.*, solution-based (wet) and vapour-based (dry) methods. Essentially, perovskite thin films are synthesized using two solid precursors having different material properties. One is a lead halide, an inorganic material with an appropriate evaporation temperature of ∼320 °C, and the other is an alkyl amino halide, an organic material with a suitable temperature of <120 °C. The “wet” and “dry” methods are subdivided into various methods, including conventional spin coating (SC) (comprised two types, (a) one-step, (b) two-step), solvent engineering modified SC, the hot casting technique, fast deposition crystallization, and vacuum-assisted and vapour-assisted solution processes. A brief review of these methods is given in the following section.

### 2.1. Conventional SC (one-step deposition)

One-step spin coating is a relatively more common method for perovskite thin-film deposition in PSCs, in an analogous way to that of solid-state-based spin coating (DSSCs).^21–23^ Essentially, a common perovskite (CH_3_NH_3_PbI_3_) solution is prepared by dissolving CH_3_NH_3_I and PbI_2_ at 1 : 1 (stoichiometric) or 1 : 3 (non-stoichiometric) molar ratio in an appropriate solvent such as gamma-butyrolactone (GBL), *N*,*N*-dimethylformamide (DMF), or dimethyl sulfoxide (DMSO). In a one-step SC process, the perovskite film formation undergoes two key phases, *i.e.*, evaporation of the excess solvent and crystallization of the perovskite film, where these steps occur simultaneously during the SC and subsequent annealing. It has been observed that one-step SC usually results in non-uniform perovskite film formation owing to the film shrinkage caused by the simultaneous solvent evaporation and crystallization.

**Fig. 2:**
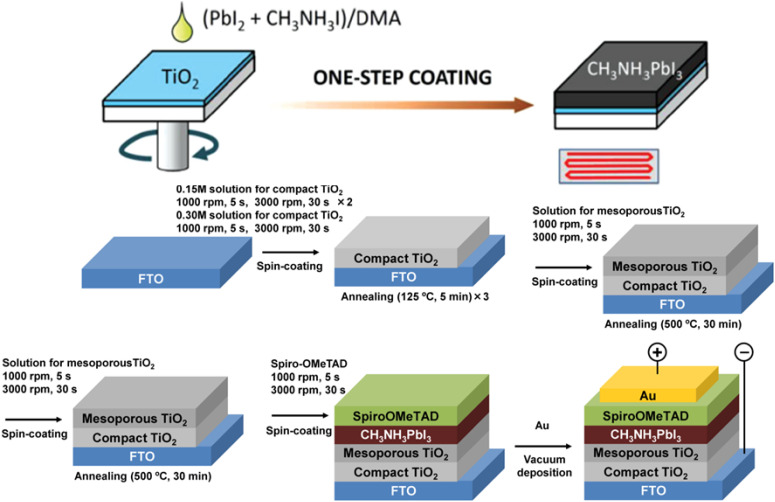
Schematic of the one-step spin coating (above) and full device configuration using one-step perovskite deposition, adapted/reproduced from ref. 27 with permission from AIP publishing, APL Mater., 2014. 2(8): p. 081510, copyright 2025.

Recently, a very high *η* of 26.10% was achieved using the spin-coating technique, where the device maintained >95% of its initial efficiency after 1090 h.^24^ Similarly, another very high *η* of 26.54% was achieved by developing an inverted PSC using the spin-coating technique.^25^ An *η* = 19.8% has been achieved using one-step SC employing a blend perovskite precursor composed of FAI, PbI_2_, MABr, and PbBr_2_ mixed in a solvent, *i.e.* a DMF and DMSO solution (volume ratio, 4 : 1) with a molar concentration of 1.35 M Pb^2+^ (PbI_2_ and PbBr_2_).^26^ The prepared solution was then spin-coated on a c-TiO_2_/FTO electrode, where it filled the pores in the TiO_2_ layer and formed a cap layer on top of the electron transport layer (ETL). A schematic of the one-step SC and full device fabrication is illustrated in Fig. 2.

### 2.2. Conventional SC (two-step deposition)

**Fig. 3:**
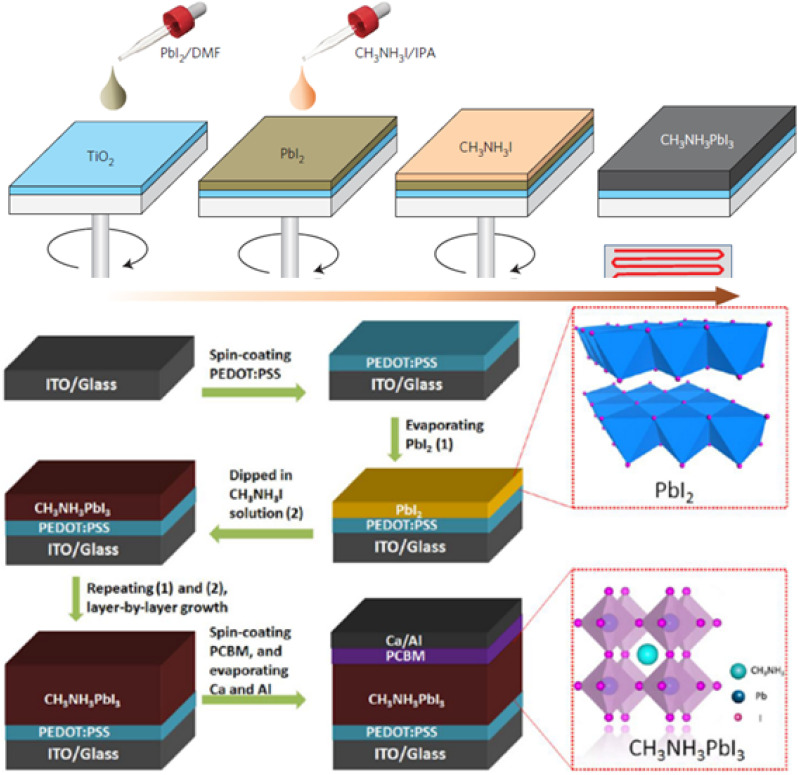
Schematic representation of the two-step spin-coating procedure for the formation of a CH_3_NH_3_PbI_3_ layer (above) and full device diagram using the two-step deposition method, adapted/reproduced from ref. 27 with permission from AIP publishing, APL Mater., 2014. 2(8): p. 081510, copyright 2025.

It has been discussed that in one-step SC, the deposition of the perovskite (a mixture of PbI_2_ and CH_3_NH_3_I dissolved in a common solvent DMF) onto *m*-TiO_2_ films results in substantial morphological variations due to the uncontrolled precipitation of the perovskite, affecting the PV performance in the devices.^28^ Conversely, two-step deposition is a useful technique that has shown remarkable improvement in high-performance devices where the perovskite can easily reach the pores in the *m*-TiO_2_.^28,29^ The two-step SC method comprises several stages: (i) dropping PbI_2_ solution onto the substrate (FTO, TiO_2_ or others), (ii) SC and annealing of PbI_2_, (iii) dipping or exposing the final prepared PbI_2_ electrode into the MAI solution/vapours, and finally annealing of the MAPbI_3_ perovskite layer. A schematic of the two-step SC procedure and full device construction is shown in Fig. 3.

Two-step deposition enables better control over the perovskite crystallization by separating the perovskite deposition into the two precursors,^28^ and changing the concentration of CH_3_NH_3_I in the two-step method greatly influences the perovskite crystal size.^30^ Im *et al.*^27^ reported that the two-step process yielded better PV performance as compared to the one-step analogue. Different parameters influencing the two-step deposition of the perovskite layer in the PSCs include: spin velocity, dipping time, annealing temperature, and CH_3_NH_3_I concentration.^31^ It has been reported that at 500 rpm, the resulting perovskite layer contained pinholes and did not fully cover the substrate. As the spin velocity increased to 2000, 4500, and 6500 rpm, the PbI_2_ films were much more uniform as compared to the slowly spun (500 rpm) samples. Additionally, fast spin coating caused an increase in the crystal size. Similarly, increasing the dipping time notably affected the crystal size, enabling the perovskite crystal to grow up to several μm.

### 2.3. Solvent engineering modified SC

**Fig. 4:**
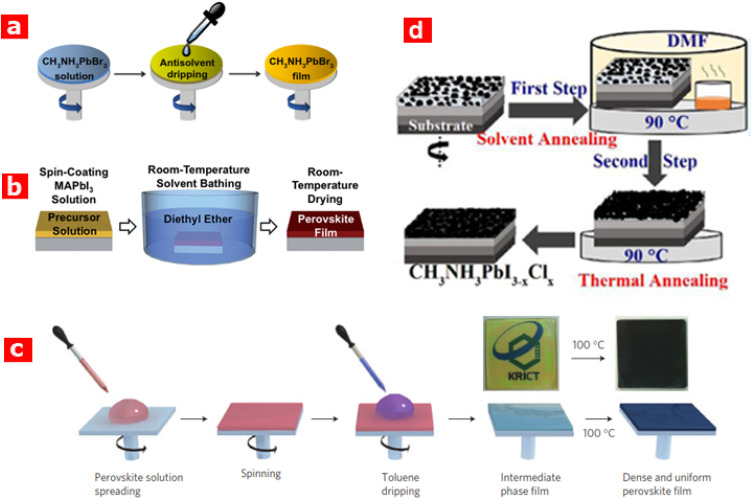
Schematic diagram of (a) anti-solvent-assisted crystallization (ASAC) for thin film CH_3_NH_3_PbBr_3_ deposition, adapted/reproduced from ref. 38, with permission from Elsevier publishing, Nano Energy, 2015. 17: p. 269–278, copyright 2025; (b) the solvent–solvent extraction (SSE) process for room-temperature perovskite film deposition, adapted/reproduced from ref. 40, with permission from RSC publishing, Journal of Materials Chemistry A, 2015. 3(15): p. 8178–8184, copyright 2025; (c) the solvent engineering procedure, adapted/reproduced from ref. 41, with permission from Nature Publishing, Nat Mater, 2014. 13(9): p. 897–903, copyright 2025; and (d) the two-step solvent-assisted annealing (TSA) process, adapted/reproduced from ref. 29, with permission from ACS publishing, ACS Applied Materials & Interfaces, 2015. 7(30): p. 16330–16337, copyright 2025.

The solvent plays an important role in the crystallization and hence influences the kinetics of crystallization and surface morphology.^32^ Adding an anti-solvent (a liquid in which the solute is insoluble) to the solution is one of the ways to decrease the solubility of the solute and subsequently achieve the state of supersaturation.^33–36^ The basic function of the anti-solvent is to reduce the solubility of the solute and subsequently accelerate the crystallization process. It is understood that CH_3_NH_3_PbI_3_ perovskites are hydrophilic in nature and are insoluble in polar solvents. As SC is one of the cheapest perovskite thin-film fabrication techniques, and the self-induced crystallization of the perovskite occurs during the SC because of the strong ionic interactions between the metal cations and halogen anions. Despite the mentioned advantages, the SC approach has shown less success in achieving homogeneous pinhole-free uniform layers over a large area.^37^ Conversely, dripping of the solvent during the SC process significantly improves the morphology of the perovskite layer. Zheng *et al.*^38^ reported the production of an extremely homogenous, packed and pinhole-free thin perovskite CH_3_NH_3_PbBr_3_ film at room temperature employing the anti-solvent-assisted crystallization (ASAC) technique (Fig. 4a). In this method, the perovskite is deposited *via* SC while dripping the anti-solvents of dichloromethane (DCM), chlorobenzene (CB) and toluene (TL) simultaneously. This approach notably enhances the PV performance of CH_3_NH_3_PbBr_3_-based devices (to *η* ≈ 8.29% and *V*_oc_ ≈ 1.42 V) *versus* conventional SC (*η* ≈ 3.15% and *V*_oc_ ≈ 1.01 V). Such results make the ASAC approach one of the recognised techniques for solidification.^39^

Similarly, the room temperature solvent–solvent extraction (SES) approach has also been successfully employed in the fabrication of perovskite thin films over a large area.^40^ In this technique, perovskite precursors initially undergo SC onto a substrate followed by immediate dipping into another solvent at room temperature (Fig. 4b). The colour of the spin-coated CH_3_NH_3_PbI_3_ substrate changes when dipped into a diethyl ether (DEE) bath, as the *N*-methyl-2-2-pyrrolidone (NMP) solvent is extracted and the perovskite's crystallization completes simultaneously within a short time (two minutes). The lower boiling point of DEE (35 °C) easily allows the residual amount on the substrate to evaporate at ambient temperature. Using the SES technique, *η* ≈ 15.2% has been achieved with a ≈ 250 nm thick perovskite film. Meanwhile, in one-step SC, usually MAI and PbX_3_, *X* = I, Cl or Br, are dissolved in a polar solvent such as DMSO, DMF, γ-butyrolactone (GBL), or NMP; therefore, it requires annealing at temperatures 70–150 °C above the boiling point of the given polar solvent. Consequently, difficulties are encountered in controlling perovskite crystallization directly from the solutions at elevated temperatures.

For instance, Jeon *et al.*^41^ achieved a highly uniform perovskite film dissolved in DMSO and GBL by dripping toluene solvent during the SC process as shown in Fig. 4c. At the initial SC stage, the film comprised perovskite precursors (PbI_2_ and MAI) dissolved in a DMSO/GBL mixture, followed by evaporation of GBL at the intermediate stage. The addition of toluene expedited the evaporation of residual DMF and formed the MAI-PbI_2_-DMF phase. The DMSO plays a significant role in retarding the reaction between MAI and PbI_2_ during the evaporation of the solvent. Finally, the resulting film was annealed at 100 °C, and a significantly high average *η* ≈ 16.46% was achieved by the solvent engineering method.

Guo *et al.*^42^ used another approach for perovskite thin-film fabrication, termed close space sublimation (CSS) deposition. In this method, a high-quality perovskite was grown over a large area (≈100 mm^2^) under a very low vacuum or even in a non-vacuum oven. In the CSS deposition method, PbI_2_ is spin coated onto a c-TiO_2_/FTO substrate as one source while MAI was spin coated onto bare glass as another source. The two coated electrodes were positioned face to face with a thin hollow aluminium foil gasket to create a closed space in an isothermal vacuum—or even non-vacuum—oven. By increasing the temperature to 150 °C in the oven, the CH_3_NH_3_I-coated film transforms into CH_3_NH_3_I vapours, and consequently reacts with the pre-deposited PbI_2_ electrode and forms CH_3_NH_3_PbI_3_. Herein, the reaction can be easily controlled by altering the CH_3_NH_3_I film thickness and reaction time, which is advantageous for the growth of perovskite films over relatively larger areas.

Liu *et al.*^29^ used a different approach by employing a new two-step solvent-assisted annealing (TSA) technique and synthesised CH_3_NH_3_PbI_3−*x*_Cl_*x*_ films. This method involved SC deposition of the mixed perovskite followed by a two-step annealing process, as shown in Fig. 4d. The first annealing was achieved by a solvent-induced process in the presence of DMF vapour in order to stimulate migration and inter-diffusion of the solvent-assisted precursor ions and molecules, enabling large-size grain growth. The solvent vapour environment treatment enables the precursor ions and molecules to diffuse a relatively longer distance, thereby increasing the grain size and film homogeneity of the perovskite.^43^ A second annealing was applied to further improve the film morphology and crystallinity. The resulting perovskite comprised grains of up to 1.1 μm in size using the TSA technique and yielded a significantly high *η* of 14% in the PSCs.

### 2.4. Hot casting technique

Besides the high *η* >20% of the PSCs, the low-cost solution processability of the perovskite thin films makes them relatively more attractive. The solution-based hot casting process is another innovative approach to produce crystalline perovskites with a significantly large grain size on the order of millimetres. The perovskite films' morphology and surface coverage are vital for device performance and could be controlled by the crystal type, nucleation and growth rate.^44^ In addition, thermal energy also plays a vital role in regulating the crystal growth. Nie *et al.*^45^ demonstrated the solution-based hot casting technique for the first time, where a continuous pin hole-free perovskite was grown with a crystal size in the range of millimetres. In this method, a hot (≈70 °C) mixture of PbI_2_ and MACl is cast onto a substrate (≈80 °C) and spin coated simultaneously. It has been shown that the perovskite crystal grain size increases significantly either by increasing the substrate temperature or utilizing a solvent with a high boiling point (DMF and NMP). The grain size obtained by the hot casting method is in the range of 1–2 mm, where the typical grain size obtained *via* conventional SC is about 1 to 2 μm. The presence of a solvent (especially with a high boiling point) on the substrate above the crystallization temperature provides a prolonged time for perovskite crystal growth and thereby results in large crystalline grains.

**Fig. 5:**
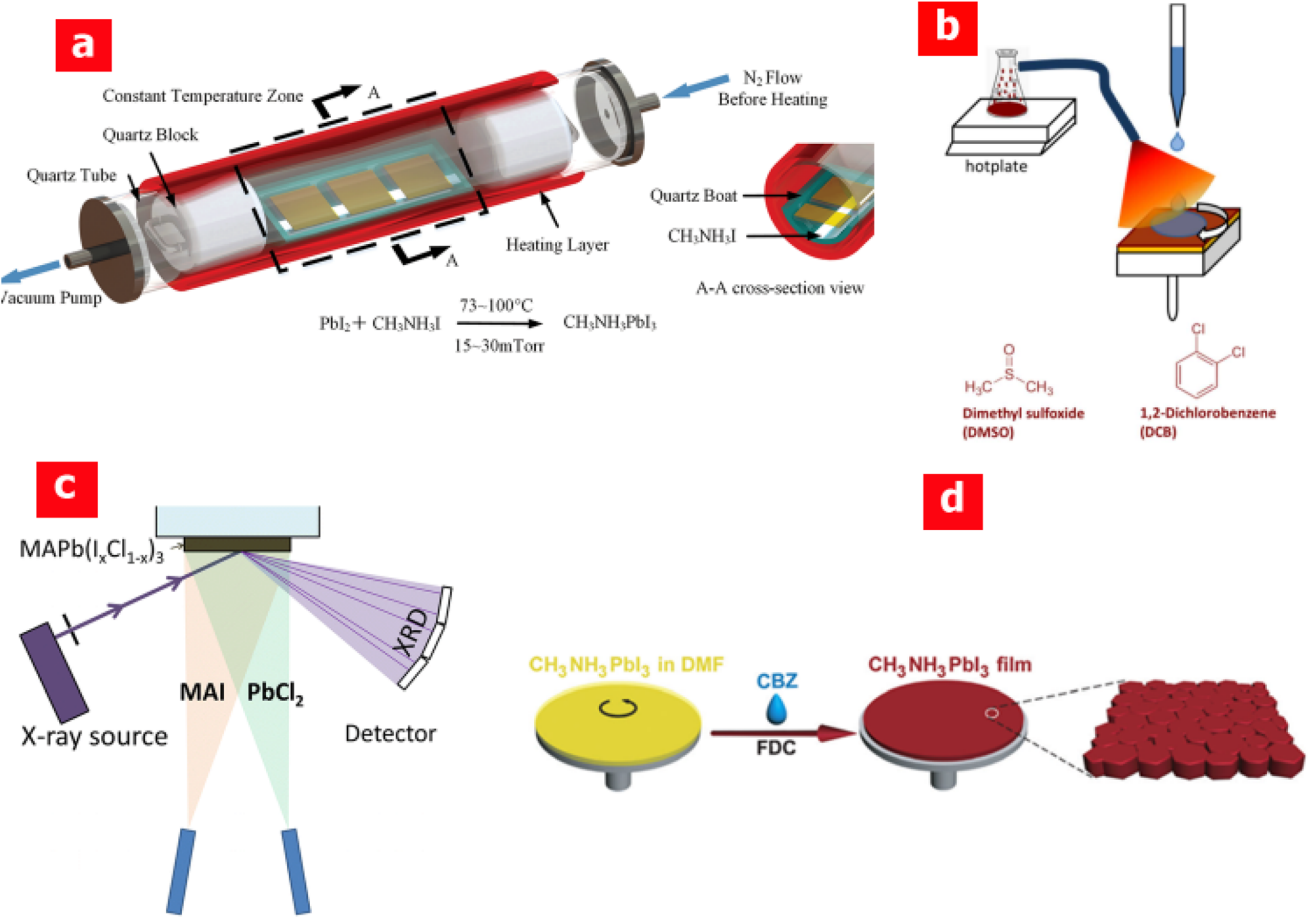
Schematic illustration of (a) hybrid physical-chemical vapour deposition (HPCVD), adapted/reproduced from ref. 47 with permission from RSC publishing, Journal of Materials Chemistry A, 2015. 3(23): p. 12436–12442, copyright 2025; (b) the solvent spin-coating process where the solvent vapour is induced by heating DMSO and DCB, adapted/reproduced from ref. 48 with permission from RSC publishing, Journal of Materials Chemistry A, 2015. 3(17): p. 9146–9151, copyright 2025; (c) the vacuum-based co-evaporation approach using two sources, adapted/reproduced from ref. 49 with permission from ACS publishing, The Journal of Physical Chemistry Letters, 2014. 5(19): p. 3308–3312, copyright 2025; and (d) fast deposition crystallization (FDC) for perovskite thin-film fabrication, adapted/reproduced from ref. 50 with permission from Wiley publishing, Angewandte Chemie, 2014. 126(37): p. 10056–10061, copyright 2025.

Zheng *et al.*^46^ reported studies regarding the thermal effect and the subsequent crystallization of CH_3_NH_3_PbI_3_ crystals using a hot casting method. They demonstrated that thermal energy plays a key role in regulating the surface morphology rather than the centrifugal force during the SC process. Peng *et al.*^47^ synthesized perovskite thin films using a hybrid physical-chemical vapor deposition (HPCVD) method under vacuum and in an isothermal environment. As evident from Fig. 5a, PbI_2_ is deposited on a *m*-TiO_2_/c-TiO_2_/FTO substrate while the CH_3_NH_3_I solid precursor is placed in a quartz boat inside an isothermal vacuum quartz tube. Heating the quartz boat produces CH_3_NH_3_I vapours that react with PbI_2_, leading to the formation of CH_3_NH_3_PbI_3_. The HPCVD method is designed to avoid any leakage from inside, as well as the ingress of any humidity or contamination. In addition, in the HPCVD technique, the vacuum level, vapour pressure and reaction temperature can be easily regulated through proper adjustment to form a highly fine perovskite film at low temperature (≈70 °C). Employing HPCVD, the best reported *η* is ≈14.7% at a reaction temperature as low as 82 °C.

Lian *et al.*^48^ employed a solvent vapor modulated spin-coating process by introducing 1,2-dichlorobenzene (DCB) and DMSO vapours to an already prepared MAPbI_3_ thin film by SC as shown in Fig. 5b. The DCB and DMSO vapours were produced by heating the filled flask at 100 °C, and the evaporated vapours were guided *via* a tube to the top of PbI_2_ for 15 min followed by SC of the MAI solution. The treated perovskite film with DCB and DMSO resulted in large grains with a prolonged carrier lifetime and a subsequent decrease in trap density. Pistor *et al.*^49^ established a technique to monitor changes in the formation of crystalline phases with the vacuum-based co-evaporation approach using two sources under varying deposition conditions, as shown in Fig. 5c. The crystalline perovskite phases were identified and monitored with *in situ* X-ray diffraction (XRD). For a low flux of PbCl_2_ content and unheated substrates, black MAPb(I_*x*_Cl_1−*x*_)_3_ with a high iodine content (*x* > 0.95) was produced with *E*_g_ ≈ 1.6 eV. Conversely, a high PbCl_2_ flux yielded a transparent MAPb(I_*x*_Cl_1−*x*_)_3_ phase with a higher Cl content (*x* ≈ 0.5).

### 2.5. Fast deposition crystallization

It is well-established that the conventional SC deposition technique results in large morphological variations and plenty of uncovered pin hole areas.^28^ Such undesirable morphologies arise from the slow crystallization of the perovskites due to the high boiling point of DMF (≈153 °C) as well as from the poor nucleation rate during the natural drying process. To avoid slow crystallization, Xiao *et al.*^50^ utilized a solvent-induced fast crystallization-deposition (FDC) technique that resulted in a flat and highly thin uniform CH_3_NH_3_PbI_3_ film, as shown in Fig. 5d. It is essentially a similar approach to that of the one-step SC procedure; however, in the FDC method, the fabricated wet perovskite substrate is immediately exposed to another solvent, preferably chlorobenzene (CBZ), to induce crystallization.

As mentioned above, the vapour deposition process likely increases the manufacturing cost while the two-step sequential SC deposition requires long processing times. Here, the FDC method is a modified form of one-step SC that minimizes the processing time, where the film formation can take place in a very short time (less than a min). In addition, a second solvent (*e.g.* chlorobenzene, CBZ) introduced on top of the wet perovskite film during the spin-coating process induces fast crystallization in the FDC process, which results in the formation of uniform-sized perovskite grains. Using the FDC approach, a very high *η* (≈16.2%) was achieved in planar PSCs.^50^

### 2.6. Vacuum-assisted method

In the planar PSCs, the very thin layer of CL TiO_2_, combined with the one-step coating of the perovskite, can lead to pinholes and incomplete coverage. As a result, holes in the HTL may directly recombine with electrons in CL TiO_2_. To resolve this issue, the two-step SC approach was introduced, where the final perovskite layer was free of pinholes, but its surface was rough owing to the severe reaction between PbI_2_ and CH_3_NH_3_I. Conversely, in vapour deposition, applying the same procedure as that of the two-step SC, instead of dipping the PbI_2_ prepared layer into the CH_3_NH_3_I solution, the former is exposed to the latter vapour at a very slow rate within a special arrangement. The fundamental principle involves subjecting the wet, solution-processed film to a low-pressure environment, which forces rapid solvent evaporation and induces a uniform, massive burst of nucleation. This results in dense, high-quality films while inherently protecting the sensitive material from ambient moisture during the critical crystallization phase. Techniques such as vacuum-quenching and Vacuum Flash-Assisted Solution Processing (VFASP) successfully utilize this mechanism to enhance the perovskite absorber. Furthermore, the utility of this approach has been extended to charge transport layers, particularly for flexible applications where high-temperature processing is restricted. For instance, vacuum-assisted low-temperature annealing has been employed for SnO_2_ electron transport layers; the rapid solvent removal in an oxygen-deficient environment significantly improved the film's optoelectronic properties and bending stability on heat-sensitive substrates, yielding efficiencies exceeding 20%.^51^ Ultimately, as emphasized in recent comprehensive reviews, integrating vacuum-based techniques with solution processing represents a critical pathway toward achieving the record-breaking efficiencies and industrial scalability required for the future of PSC technology.^16^

### 2.7. Vapour-assisted solution process (VASP)

Producing high-quality perovskite thin films is a prerequisite in the fabrication of PSCs like other thin-film solar technologies, such as CdTe, a-Si, and CuGaInS_2_. Vacuum evaporation is one of the promising techniques in perovskite thin-film fabrication and has shown promising results in planar PSCs. However, this technique requires a high vacuum, which is highly energy-intensive and therefore, unsuitable for large-scale production.^52^ Conversely, solution-based processes have also been suggested to fabricate thin films; however, these result in the formation of pinholes and incomplete surface coverage, which worsens the film quality and thereby deteriorates the device performance. This unusual behaviour in the perovskite morphology is due to the lack of suitable solvents that can dissolve both components (MAI and PbI_2_) as well as the fast reaction of the perovskite component.^37^

**Fig. 6:**
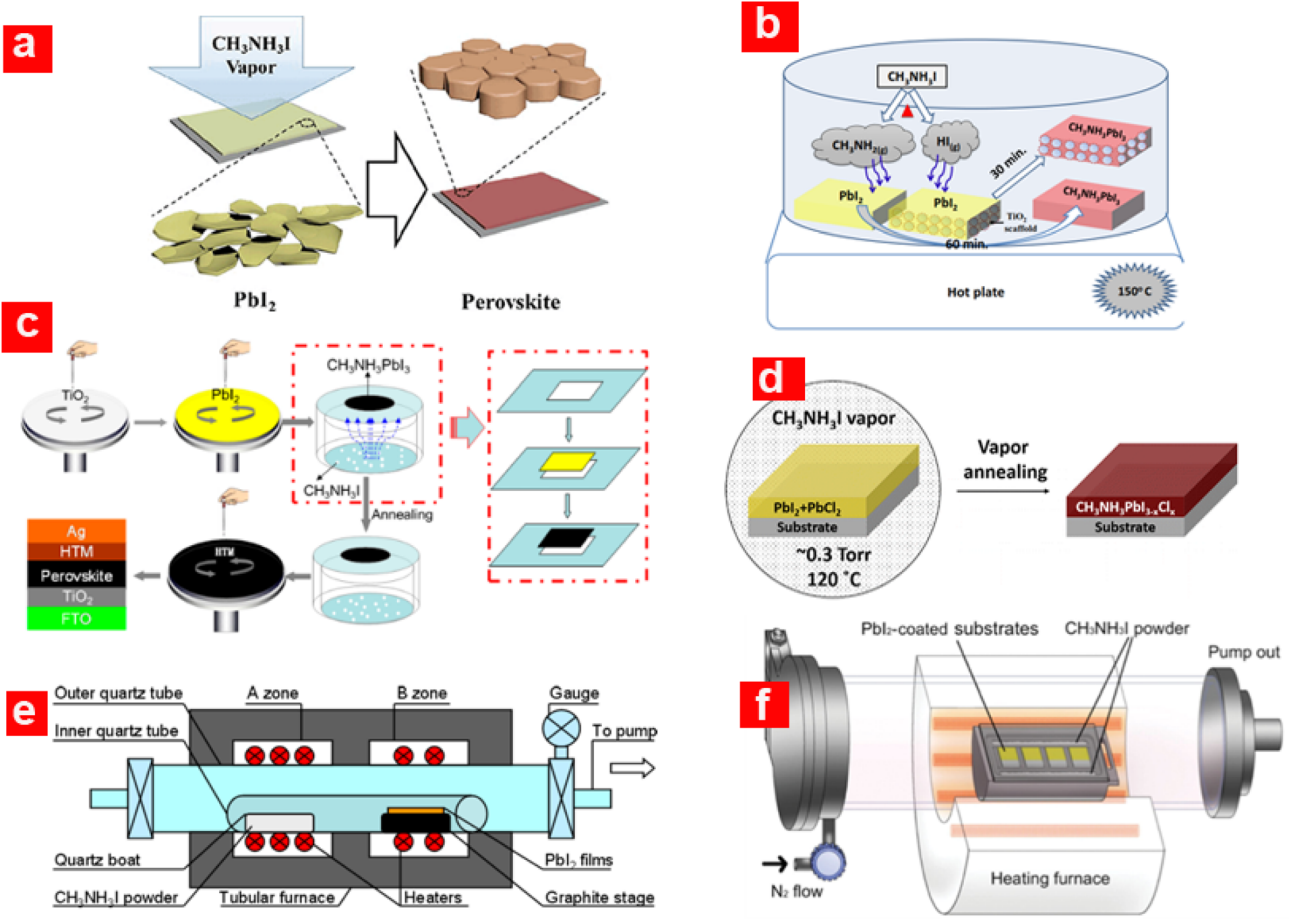
Schematic demonstration of perovskite film formation using the vapour-assisted solution process (VASP) (a) adapted/reproduced from ref. 53 with permission from ACS publishing, Journal of the American Chemical Society, 2014. 136(2): p. 622–625, copyright 2025; (b) adapted/reproduced from ref. 56 with permission from RSC publishing, Journal of Materials Chemistry A, 2016. 4(7): p. 2630–2642, copyright 2025; and (c) adapted/reproduced from ref. 60 with permission from ACS publishing, ACS Applied Materials & Interfaces, 2015. 7(17): p. 9066–9071, copyright 2025. (d) Low-pressure-VASP, adapted/reproduced from ref. 61 with permission from ACS publishing, The Journal of Physical Chemistry Letters, 2015. 6(3): p. 493–499, copyright 2025. (e) Low pressure chemical vapour deposition, adapted/reproduced from ref. 63 with permission from ACS publishing, ACS Applied Materials & Interfaces, 2015. 7(4): p. 2708–2714, copyright 2025. (f) Low pressure hybrid CVD, adapted/reproduced from ref. 67 with permission from Wiley, Advanced Materials Interfaces, 2016. 3(8): p. 1500849, copyright 2025.

The vapour-assisted solution process (VASP) is a combination of the solution and vapour deposition processes. In the VASP technique, the formation of an inorganic (PbI_2_) film takes place *via in situ* reaction by depositing it on c-TiO_2_/FTO, while immediately exposing it to the organic (CH_3_NH_3_I) vapours as shown in Fig. 6a.^53,54^ The evaporation occurs at a low temperature (∼150 °C) in a N_2_ atmosphere. With this method, the perovskite layer shows a well-defined grain structure (with a size of the micrometre) with full surface coverage having minimal roughness, which is favourable for any high PV technology. Li *et al.*^55^ employed an indigenous set-up for VASP with a low annealing temperature of up to 130 °C. The resulting perovskite grain size was in the range of ∼600 nm and delivered *η* ≈ 12.6% in PSCs. Jain *et al.*^56^ employed a similar VASP method shown in Fig. 6b for *m*-TiO_2_-based PSCs with the reaction mechanism given in eqn (1). It has been shown that an intermediate gas phase of methylamine and hydrogen iodide is formed during the conversion of PbI_2_ into CH_3_NH_3_PbI_3_

1 PbI_2_(s) + CH_3_NH_3_I(s) →PbI_2_ + CH_3_NH_2_(g) + HI(g) → CH_3_NH_3_PbI_3_(s)

The formation of a similar intermediate phase between PbI_2_ polytypes and CH_3_NH_3_PbI_3_ has also been reported by others.^57,58^ It is anticipated that the small CH_3_NH_2_ molecule and HI could intercalate relatively easily into the PbI_2_ structure, and the volume of the PbI_2_ after conversion to CH_3_NH_3_PbI_3_ increases by 100% as given in eqn (2)2
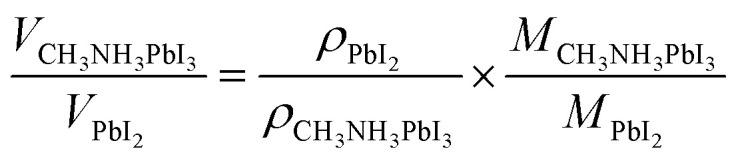
where *V* is the volume, *M* is the molecular formula weight, and *ρ* is the density, which is 4.16 g.cm^−3^ for CH_3_NH_3_PbI_3_ and 6.16 g.cm^−3^ for PbI_2_.^59^ Liu *et al.*^60^ employed a modified VASP technique with the help of culture dishes to fix the PbI_2_ substrate, which was placed face down on the CH_3_NH_3_I powder, as shown in Fig. 6c. The bottom-up CH_3_NH_3_I vapours assist and expedite the perovskite's crystallization, which results in full coverage of the surface, and the grain size increases up to a micrometre in size. Controlling the kinetic reactivity of PbI_2_ and CH_3_NH_3_I, along with the perovskite stability during the *in situ* growth process, could allow scaling up of the thin-film perovskite technology to mass production.

Li *et al.*^61^ presented a two-step low-pressure VASP (LP-VASP) technique for perovskite thin-film formation, as shown in Fig. 6d. In the first step, the mixed inorganic (PbI_2_/PbCl_2_) framework was spin-coated onto a c-TiO_2_/FTO substrate with a homogeneous surface coverage. In the second step, the prepared PbI_2_/PbCl_2_/c-TiO_2_/FTO substrate was exposed to CH_3_NH_3_I vapour at low pressure and was annealed simultaneously. The low-pressure vapour annealing was reported to enhance the vapour pressure of CH_3_NH_3_I and thereby lowered the annealing temperature to 120 °C, which was quite low compared to that (150 °C) of VASP.^53^ Planar PSCs fabricated by LP-VASP yielded a record high *η* ≈ 16.8% with a significantly reduced J-V hysteresis. A similar approach (LP-VASP technique) was utilized in another study, which reported the fabrication of a hybrid perovskite CH_3_NH_3_Pb(SCN)_*x*_I_3−*x*_ film from Pb(SCN)_2_ precursor in the CH_3_NH_3_I vapour environment.^62^ The CH_3_NH_3_Pb(SCN)_*x*_I_3−*x*_ based device yielded *η* ≈ 12.72% in comparison to *η* ≈ 11.32% for the CH_3_NH_3_PbI_3_ counterpart. The Pb(SCN)_2_ is less expensive and comprises only 4% of PbI_2_ and is, therefore, considered the best alternative for future PSC technology.

Luo *et al.*^63^ developed a homemade apparatus to perform a technique referred to as low-pressure chemical vapour deposition (LPCVD), as shown in Fig. 6e, for perovskite layer formation, which significantly slowed down the very rapid intercalating reaction rate. The LPCVD apparatus is basically adopted from a conventional double zone CVD tubular furnace, which could allow easy scaling up of the process for mass production. LPCVD is a well-established industrial technology for thin-film deposition for large-scale production of a-Si, Si_3_N_4_, SiO_2_, and ZnO.^64,65^ The SEM and AFM analyses reveal that the LPCVD approach produces better morphology, super-uniform, homogeneous and well-defined CH_3_NH_3_PbI_3_ films. Using the LPCVD approach, the best device yielded *η* of 12.73% with CH_3_NH_3_PbI_3_ grain size of 500 nm. Another modified version of VASP is the low temperature vapour-assisted solution process (LT-VASP).^66^ The LT-VASP technique was employed to compare SnI_2_ and PbI_2_ precursor-based perovskites, where it was shown that the former reacts much faster with MAI than the latter. The black phase of the perovskite is formed in merely 1 min using the LT-VASP technique, while VASP takes about 2 h to completely transform PbI_2_ into CH_3_NH_3_PbI_3_. This astonishing result suggests that MAI gas diffuses rapidly into SnI_2_, and that the Gibbs free energy for forming CH_3_NH_3_SnI_3_ is lower than that for forming CH_3_NH_3_PbI_3_ from PbI_2_. From the industrial point of view, the fabrication of thin-film perovskite based on Sn using LT-VASP could make it a highly practical approach for solar cell manufacturing.

Impressed by the vapour-based deposition (*i.e.*, co-evaporation deposition and LP-VASP techniques) which yielded high-quality and uniform perovskite films, Shen *et al.*^67^ applied the low-pressure hybrid chemical vapour deposition (LPHCVD) technique for perovskite thin-film fabrication. The LPHCVD process occurs in a quartz tube where the PbI_2_/c-TiO_2_/FTO substrate is placed in a capped graphite boat to ensure a uniform heating environment for the vapour (Fig. 6f). The final perovskite formed by the MAI vapour was kept inside the graphite box and heated at a certain temperature and pressure to produce the MAI vapour. The LPHCVD technique has more advantages over the aforementioned methods due to the high material yield ratio and large-area scalable capabilities. Using the LPHCDV technique for perovskite deposition, the mesoporous and planar PSCs yielded *η* ∼ 14.99% and 15.37%, respectively.

To provide a clear and comparative overview of the advancements discussed, the performance of perovskite solar cells fabricated using various key deposition techniques is summarized in Table 1. This table shows the device structures, notable features, and corresponding power conversion efficiencies, highlighting the progress achieved through different fabrication strategies.

**Table 1:** Summary of the performance of perovskite solar cells fabricated by various deposition techniques

Deposition technique	Device structure	PCE (%)	Reference
One-step spin coating	Inverted (p-i-n) ITO/HTL/Perovskite/ETL/Ag	26.54%	[25]
One-step spin coating	Regular (n-i-p) FTO/ETL/Perovskite/HTL/Au	26.10%	[24]
Solvent engineering (SES)	Regular (n-i-p) FTO/TiO_2_/Perovskite/HTL/Au	∼15.2%	[40]
Solvent engineering (toluene)	Regular (n-i-p) FTO/TiO_2_/Perovskite/HTL/Au	∼16.46%	[41]
Hot casting	Regular (n-i-p) FTO/ETL/Perovskite/HTL/Au	>20%	[44]
Fast deposition (FDC)	Regular (n-i-p) planar FTO/TiO_2_/Perovskite/HTL/Au	∼16.2%	[50]
Hybrid CVD (HPCVD)	Regular (n-i-p) FTO/TiO_2_/Perovskite/HTL/Au	∼14.7%	[47]
Vapour-assisted (VASP)	Regular (n-i-p) FTO/TiO_2_/Perovskite/HTL/Au	∼12.6%	[55]
Low-pressure VASP (LP-VASP)	Regular (n-i-p) planar FTO/TiO_2_/Perovskite/HTL/Au	∼16.8%	[61]

### 2.8. Deposition strategies for all-inorganic perovskites

A significant challenge for the long-term stability of PSCs is the volatile and thermally sensitive nature of organic cations. To address this, all-inorganic perovskites, including cesium (*e.g.*, CsPbI_3_, CsPbBr_3_), have garnered immense attention for their superior intrinsic thermal stability. However, the fabrication of high-performance all-inorganic perovskites presents a unique challenge, *i.e.*, the desirable photoactive black perovskite phase (α-phase) is often metastable at room temperature and can readily transform into a photo-inactive yellow δ-phase. Therefore, thin-film deposition and fabrication strategies are crucial for both forming and stabilizing the α-phase. Recent advancements have focused on two critical areas, namely controlling the perovskite crystallization and engineering the interfaces. For crystallization control, solution-based deposition methods have been refined with novel strategies. For instance, a multistep solution-processing approach has been developed to precisely tune the deposition and control the phase conversion, leading to high-purity CsPbBr_3_ films with excellent stability and high *η* in PSCs without a dedicated hole-transporting layer.^68^ Another powerful solution-based strategy is additive engineering, where trace amounts of impurity ions like Cd^2+^, are incorporated into the CsPbIBr_2_ precursor solution. This simple and effective method has been shown to improve the film crystallinity, reduce the trap density, and optimize the energy levels, significantly boosting the final device *η* and stability.^69^

Complementing these efforts, interface engineering has proven to be a direct and highly effective way to enhance performance. On the electron transport side, novel composite ETLs, such as TiO_2_ nanoparticles embedded in functionalized MXene, have been shown to reinforce the ETL/perovskite contact, release lattice strain, and suppress non-radiative recombination, leading to a champion *η* of over 15% in carbon-based CsPbI_2_Br PSCs.^70^ On the charge-collecting electrode side, constructing a heterojunction thin layer, such as PbS/CdS, between the perovskite and the carbon electrode can effectively reduce the trap density, inhibit ion migration, and improve moisture resistance, leading to devices with remarkable stability and high *V*_oc_.^71^ These combined strategies in deposition and device engineering are crucial for unlocking the full potential of all-inorganic perovskites as a more durable alternative for next-generation photovoltaics.

## 3 Conclusions

Most of the perovskite solar cells (PSCs) are fabricated at a lab scale with cell dimensions in cm^2^. PSCs are fabricated by stacking layer-by-layer materials which required *via* various deposition techniques. Recent advances in deposition techniques for perovskite solar cells (PSC) have drastically enhanced the performance of PSCs in terms of power conversion efficiency (*η*) and stability. These deposition techniques have significantly improved thin-film quality, device performance, and process scalability. Among various deposition techniques, spin coating has yielded the highest *η* and stable performance in PSCs. Advancements in deposition techniques targeting long-term stability, large-scale production, and environmentally sustainable processing remain a critical challenge for the commercialization of PSCs. Future efforts should focus on hybrid deposition strategies, defect-tolerant formulations, and roll-to-roll compatible processes that combine high throughput with precise morphology control. With continued innovation in deposition technology, perovskite solar cells are poised to transition from laboratory prototypes to a competitive renewable energy solution.

## Author contributions

Qamar Wali: conceptualization, investigation, writing – original draft. Nahin Ar Rabbani: investigation, software. Sadia Afrin: investigation. It Ee Lee: supervision, reviewing and editing, project administration, funding acquisition: Mohammad Yar Khan: supervision, writing – review & editing. Muhammad Aamir: supervision and writing – review & editing.

## Conflicts of interest

There are no conflicts to declare.

## Data Availability

For this review article, no new data were generated or analyzed.
